# Inhibition of macrophage inflammasome assembly and pyroptosis with GC-1 ameliorates acute lung injury

**DOI:** 10.7150/thno.101866

**Published:** 2025-01-20

**Authors:** Bin Li, Jingyi Liu, Wanyu He, Yanlin Zhou, Man Zhao, Cong Xia, Xiaoyue Pan, Zhihua Ji, Ruoyu Duan, Hui Lian, Kai Xu, Guoying Yu, Lan Wang

**Affiliations:** State Key Laboratory of Cell Differentiation and Regulation, Henan International Joint Laboratory of Pulmonary Fibrosis, Henan Center for Outstanding Overseas Scientists of Organ Fibrosis, Pingyuan Laboratory, College of Life Science, Henan Normal university, Xinxiang 453007, China.

**Keywords:** GC-1, ALI/ARDS, macrophage, pyroptosis, Nrf2-p53-ASC pathway

## Abstract

**Rationale:** Acute lung injury (ALI)/acute respiratory distress syndrome (ARDS) is a critical syndrome with a mortality rate of up to 40%, and it is characterized by a prominent inflammatory cascade. The inflammasome and pyroptosis play crucial regulatory roles in regulating various inflammatory-related diseases by serving as pivotal signaling platforms for inflammatory responses and mediating the release of substantial quantities of inflammatory factors. Our previous studies confirmed that GC-1, a clinical-stage thyroid hormone analog, effectively mitigated pulmonary fibrosis by restoring mitochondrial function in epithelial cells. However, the potential effects of GC-1 on macrophage inflammasome assembly and pyroptosis in lung injury as well as the underlying mechanisms, remain unclear.

**Methods:** The effects of GC-1 on lung injury, oxidative damage and inflammation were evaluated in two murine models of ALI (LPS- or HCl-induced models) by assessing lung pathology, the concentrations of IL-1β and IL-18 in BAL fluid, inflammasome and the levels of inflammasome- and pyroptosis-related proteins. Additionally, the impact of GC-1 on ROS-mediated inflammasome assembly and pyroptosis was investigated by examining ROS levels, Nrf2 signaling, and inflammasome adaptor protein ASC levels in mouse alveolar macrophages and human THP-1 macrophages treated with LPS and ATP. The Nrf2 inhibitor ML385 and the mitochondrial-ROS inhibitor Mito-TEMPO were used to further elucidate the effect of GC-1 on the Nrf2-p53-ASC pathway.

**Results:** GC-1 significantly alleviated inflammation and lung injury in ALI model mice, as indicated by pulmonary pathology, inflammatory cytokine levels, ROS production and pyroptosis rates. Consistently, GC-1 inhibited ASC recruitment and oligomerization in macrophages, which suppressed the gasdermin D-mediated release of IL-1β and IL-18. These findings indicated a reduction in inflammasome assembly and pyroptosis initiation. Further research revealed that GC-1 may mitigate oxidative stress induced by mitochondrial damage through Nrf2 signaling, thereby inhibiting the expression of ROS-activated p53 and the target gene ASC. This protective effect of GC-1 could be reversed by ML385 and mimicked by Mito-TEMPO.

**Conclusions:** This study presents a novel mechanism for treating ALI in which GC-1 inhibits macrophage ROS-mediated inflammasome assembly and pyroptosis through Nrf2-p53-ASC pathway. These findings highlight the promising potential of the use of GC-1 as an anti-inflammatory and antioxidant drug in the treatment of ALI/ARDS.

## Introduction

Acute lung injury/acute respiratory distress syndrome (ALI/ARDS) is a severe respiratory condition triggered by various factors, such as sepsis, pancreatitis, and gastric content aspiration 1. There are limited treatment options for ARDS, and it accounts for 10% of ICU admissions, with a mortality rate of up to 40% 2. A retrospective cohort study of patients with ARDS reported 90-day mortality rates of 19% in pediatric cohorts, 33% in adult trials, and 67% in adult observational cohorts 3. The global impact of COVID-19 in 2019 highlighted the heterogeneous and critical nature of ALI/ARDS 4. Targeting the molecular characteristics of ARDS and implementing personalized medicine strategies may help effectively treat ARDS 5.

Lytic cell death (e.g. pyroptosis and necroptosis) induces inflammation and disease through the release of inflammatory factors and intracellular components 6. The inflammasome, a central signaling platform for inflammatory responses induced by pathogen infection and tissue damage, regulates various inflammation-related diseases 7. In the inflammasome complex, the adaptor protein apoptosis-associated speck-like protein containing a CARD (ASC) oligomerizes in response to sensors such as NLR family pyrin domain-containing 3 (NLRP3), recruiting and activating caspase-1. This leads to gasdermin D cleavage and pore formation, resulting in pyroptosis and the release of mature isoforms of IL-1β and IL-18 through active caspase-1 cleavage 8, 9. Inhibiting inflammasome activation and pyroptosis is a potential strategy for treating ALI/ARDS 10.

The accumulation of intracellular reactive oxygen species (ROS) exacerbates the inflammatory response in ALI/ARDS 11, 12. Nuclear factor erythroid 2-related factor 2 (Nrf2) is a key component involved in antioxidant responses and cellular protection 13. p53 and Nrf2 respond differently to ROS, regulating oxidative stress. Under low-ROS, p53 upregulates the expression of antioxidant genes for protection. However, excessive ROS levels increase p53 expression levels, which inhibits Nrf2-mediated antioxidant signaling and triggers p53-mediated apoptosis 14, 15. The regulation of ROS levels and Nrf2 signaling is crucial for protection against ALI/ARDS 10, 16, 17. Our previous studies confirmed that GC-1 can effectively restore mitochondrial function in alveolar epithelial cells through the PINK1 pathway 18, 19. These findings strongly suggest that GC-1 may have the potential to treat ALI by reducing the accumulation of ROS caused by mitochondrial damage.

Thyroid hormones (THs) play important roles in development and metabolism 20. GC-1 (Sobetirome), a selective TH receptor β1 (TRβ1) agonist, mimics T_3_ without causing cardiac side effects. It shows promise in the treatment of dyslipidemia, hepatocellular carcinoma, and demyelinating diseases 21. The efficacy and safety of GC-1 in reducing low-density lipoprotein cholesterol have been demonstrated in phase I clinical trials 21. Studies have confirmed that THs can improve lung function and surfactant balance, as well as reduce inflammation, thereby alleviating lung injury in mice 22-24. Our previous results also indicate that T_3_ or GC-1 can mitigate pulmonary fibrosis by improving epithelial mitochondrial function 18, 19. However, the mechanisms by which GC-1 protects against pulmonary inflammation induced by macrophage pyroptosis in ALI remain unclear.

This study aimed to investigate the therapeutic effects and underlying mechanism of GC-1 in ALI/ARDS through the inhibition of macrophage inflammasome assembly and pyroptosis. We evaluated lung tissue pathology, oxidative damage and inflammatory factors in LPS- or HCl-induced mouse models. Additionally, we investigated the impact of GC-1 on ROS-induced inflammasome assembly by examining changes in Nrf2 signaling, ROS levels, and the levels of proteins related to the inflammasome and pyroptosis in mouse alveolar macrophages (AMs) and human THP-1 macrophages that were treated with LPS and ATP. Finally, we revealed a mechanism for treating ALI in which GC-1 inhibits macrophage ROS-mediated inflammasome activation and pyroptosis through the Nrf2-p53-ASC pathway.

## Results

### GC-1 alleviated LPS- or HCl-induced ALI and pyroptosis in mice

**Figure 1:**
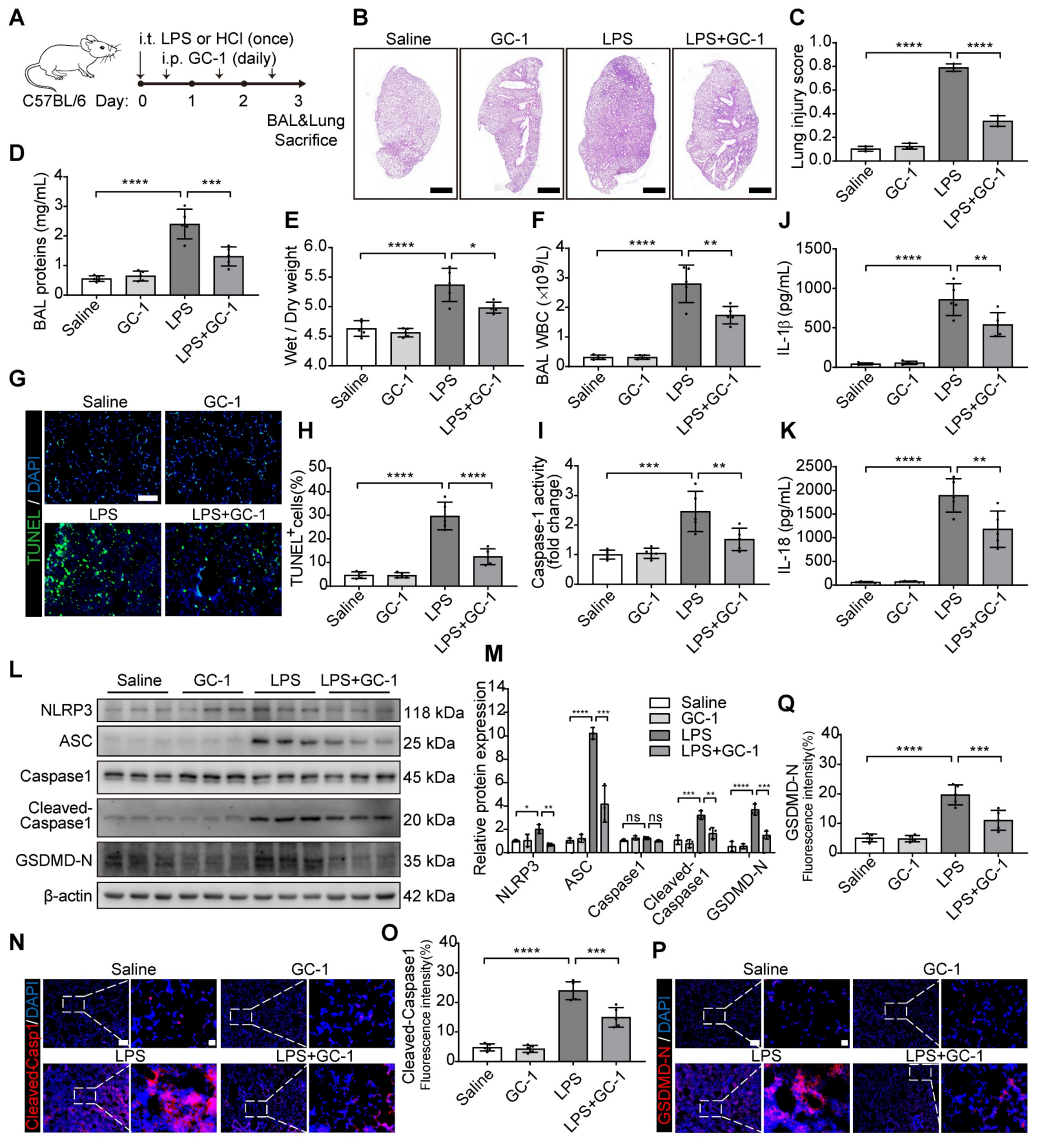
** GC-1 alleviated LPS-induced lung injury and pyroptosis in mice.** C57BL/6 mice were intratracheally instilled with LPS (5 mg/kg body weight) or HCl (0.1 M, pH 1.0) on day 0 to induce ALI, followed by daily intraperitoneal injections of GC-1 (100 µg/kg body weight) 12 h later for 3 consecutive days. (**A**) Schematic representation of the experiment. (**B**) Representative paraffin sections of lung tissue from LPS-induced mice (n = 3), H&E staining, Scale bars, 1000 µm. (**C**) Lung injury score of LPS-induced ALI according to the American Thoracic Society (ATS) lung injury scoring system (n = 3). (**D**) BAL protein content, (**E**) lung wet/dry weight ratio, (**F**) BAL white blood cell (WBC) concentration for evaluating lung injury in mice induced by LPS (n = 5). (**G**) Representative frozen sections (n = 5), TUNEL staining, Scale bars, 100 µm. (**H**) Quantitative statistical results of TUNEL-stained positive cells (n = 5). (**I**) Caspase-1 activity in lung tissue, normalized to saline group (n = 5). ELISA detection of (**J**) IL-1β and (**K**) IL-18 levels in mouse BAL (n = 5). (**L**) WB analysis of NLRP3, ASC, Capase1, Cleaved-Capase1, and GSDMD-N expression in mouse lung, with β-actin as reference (n = 3). (**M**) Quantitative analysis of immunoblot grayscale values (n = 3). Immunofluorescence (IF) staining of lung tissue showing (**N**) Cleaved-Capase1 and (**P**) GSDMD-N representative images (n = 5), Scale bars, 100 µm and 20 µm (insets), and quantitative statistical results of fluorescence intensity from (**O**) Cleaved-Capase1 and (**Q**) GSDMD-N with DAPI as reference (n = 5). ASC: apoptosis-associated speck-like protein containing a card; BAL: bronchoalveolar lavage; GSDMD-N: n-terminal fragment of gasdermin D; NLRP3: nlr family pyrin domain-containing 3. The data represent one of at least three independent experiments. The values are shown as mean ± SD. *P < 0.05; **P < 0.01; ***P < 0.001; ****P < 0.0001.

The therapeutic potential of GC-1 in ALI was investigated by establishing two mouse models of ALI through the intratracheal instillation of LPS or HCl to simulate ALI caused by bacterial infection or gastric aspiration as reported in clinical cases. This was followed by intraperitoneal injection of GC-1 12 h later (Figure 1A). LPS or HCl induced significant lung injury, as shown by neutrophil infiltration, alveolar wall thickening, and the formation of hyaline membranes in the alveoli and interstitium; these effects were alleviated by GC-1 treatment (Figure 1B-C, Figure S1A-B). GC-1 also reduced the lung wet-to-dry weight ratio, bronchoalveolar lavage (BAL) protein concentration and white blood cell concentration (Figure 1D-F, Figure S1C-D). Furthermore, we investigated the effect of GC-1 on pyroptosis in the ALI model mice. TUNEL staining was performed to detect DNA damage, and GC-1 significantly reduced the number of lung cells that were positive for TUNEL staining in the ALI model mice (Figure 1G-H). Moreover, GC-1 inhibited the increase in pulmonary Caspase-1 enzyme activity (Figure 1I), as well as the increase in IL-1β and IL-18 concentrations in BAL fluid (Figure 1J-K). Further analysis revealed that GC-1 inhibited the LPS-induced upregulation of the pyroptosis-related proteins NLRP3, ASC, Cleaved-Caspase1, and the N-terminal fragment of gasdermin D (GSDMD-N) induced by LPS (Figure 1L-M), which indicated that GC-1 suppressed NLRP3 inflammasome activation. The immunofluorescence results also confirmed that GC-1 reduced the accumulation of Cleaved-Caspase1 and GSDMD-N in the area of the lung lesion (Figure 1N-Q). These collective findings demonstrated that GC-1 effectively alleviates LPS or HCl-induced ALI and pyroptosis in mice.

### GC-1 inhibited LPS- and ATP-induced macrophage pyroptosis

**Figure 2:**
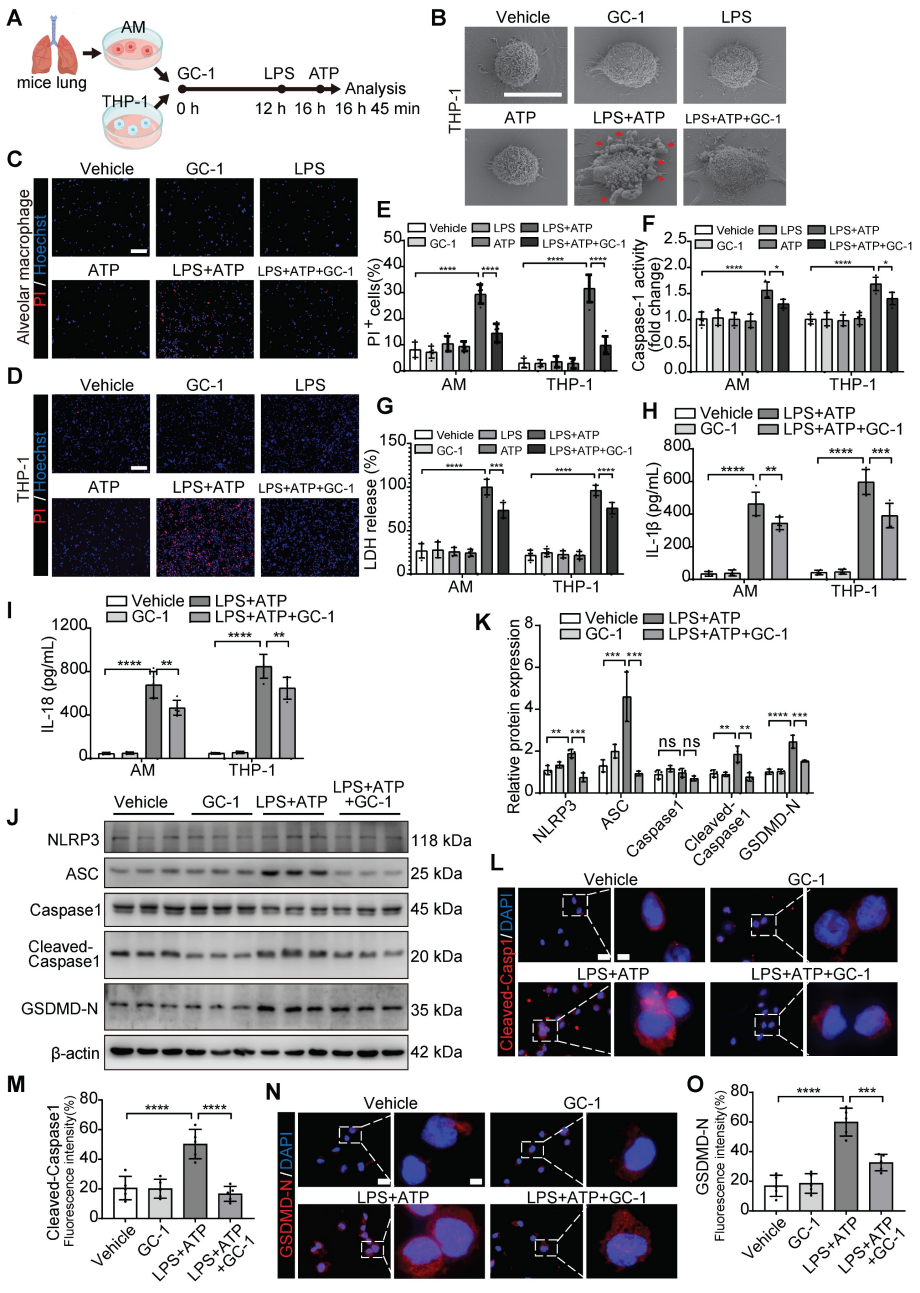
** GC-1 inhibited pyroptosis in mouse alveolar macrophages (AMs) and human THP-1 macrophages induced by LPS and ATP.** AMs or THP-1 cells were pretreated with GC-1 (100 nM) for 12 h, followed by LPS (100 ng/mL) for 4 h, and then ATP (5 mM) induction for 45 min before sample collection. (**A**) Schematic diagram. (**B**) Representative scanning electron microscopy (SEM) images of THP-1 (n = 3), with arrows indicating pyroptotic bodies, Scale bars, 10 µm. Representative images of (**C**) AMs and (**D**) THP-1 cells stained with propidium iodide (PI) and Hoechst33342 (n = 5), Scale bars, 200 µm. (**E**) Quantitative statistical results of PI-stained positive cells (n = 5). (**F**) Caspase-1 activity in AMs and THP-1 cells, normalized to the vehicle group (n = 5). (**G**) Lactate dehydrogenase (LDH) cytotoxicity in AMs and THP-1 cell supernatants, normalized to the LPS-treated group (n = 5). ELISA detection of (**H**) IL-1β and (**I**) IL-18 levels in AMs and THP-1 cell supernatants (n = 5). (**J**) Due to the low yield and limited lifespan of primary AMs from mouse BAL, WB analysis was performed exclusively in THP-1 cells, with β-actin as an internal reference (n = 3). (**K**) Quantification of immunoblot grayscale values (n = 3). Representative images of IF staining of THP-1 cells for (**L**) Cleaved-Caspase1 and (**N**) GSDMD-N (n = 5), Scale bars, 20 µm and 5 µm (insets), and quantitative statistical results of fluorescence intensity from (**M**) Cleaved-Capase1 and (**O**) GSDMD-N with DAPI as reference (n = 5). ASC: apoptosis-associated speck-like protein containing a card; GSDMD-N: n-terminal fragment of gasdermin D; LDH: lactate dehydrogenase. The data represent one of at least three independent experiments. The values are shown as mean ± SD. *P < 0.05; **P < 0.01; ***P < 0.001; ****P < 0.0001.

AMs and THP-1 macrophages were treated with LPS and ATP to investigate the inhibitory effect of GC-1 on pyroptosis (Figure 2A). Scanning electron microscopy revealed that LPS and ATP treatment caused THP-1 cells to swell, enlarge, and many bubble-like protrusions formed on the cell surface. However, GC-1 significantly reduced these typical features of pyroptosis (Figure 2B). PI staining indicated disruption of the cell membrane, and GC-1 significantly attenuated the number of PI-stained positive cells following treatment with LPS and ATP (Figure 2C-E). Additionally, GC-1 decreased the Caspase-1 enzyme activity in the lysates (Figure 2F), as well as concentrations of LDH, IL-1β, and IL-18 in the supernatants (Figure 2G-I). Collectively, these findings indicated that GC-1 alleviated cell membrane rupture induced by LPS and ATP. Further analysis of the pyroptosis-related proteins NLRP3, ASC, Cleaved-Caspase1, and GSDMD-N in THP-1 cells confirmed that GC-1 inhibited the LPS- and ATP-induced NLRP3 inflammasome activation, which reduced the production of downstream Cleaved-Caspase1 and GSDMD-N (Figure 2J-O). Taken together, these results demonstrated that GC-1 effectively inhibited LPS- and ATP-induced macrophage pyroptosis.

### GC-1 reduced mitochondrial damage and ROS levels *in vivo* and *in vitro*

**Figure 3:**
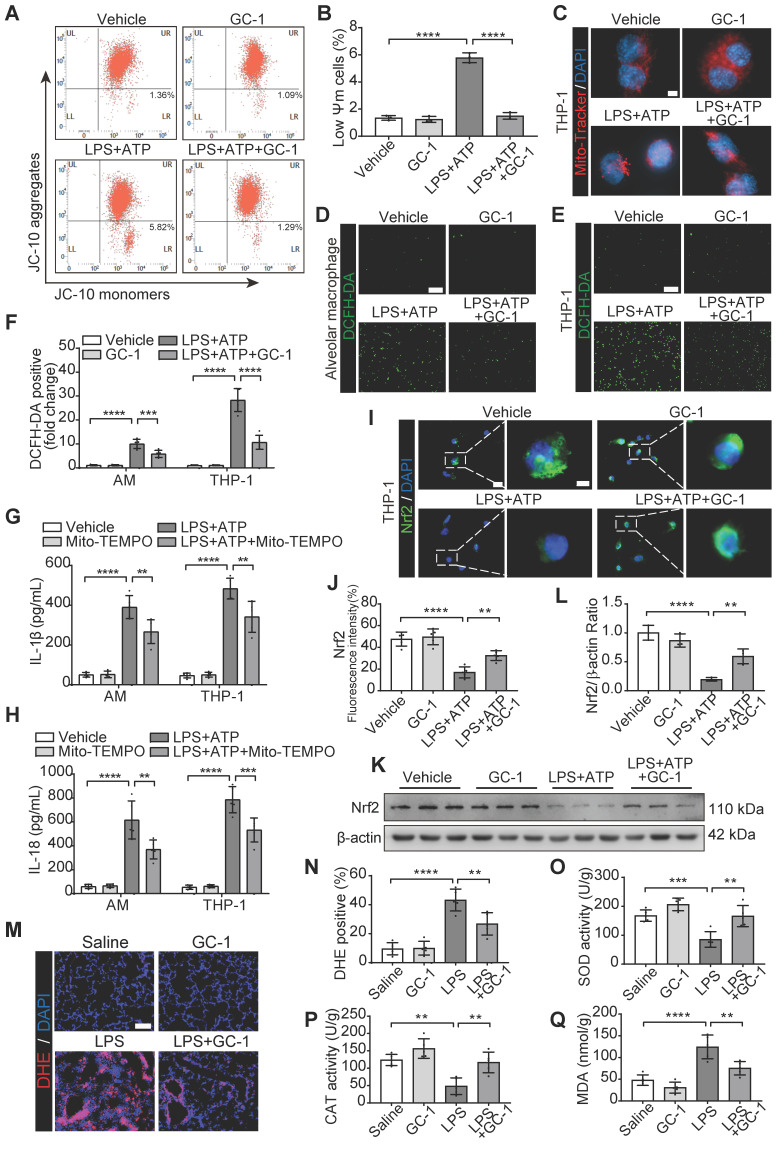
** GC-1 attenuated mitochondrial damage and oxidative stress injury.** (**A**) Representative flow cytometry images showing JC-10 probe detecting mitochondrial membrane potential in THP-1. (**B**) Quantification of mitochondrial membrane potential (n = 3). (**C**) Representative images of Mito-Tracker staining in THP-1 (n = 5), Scale bars, 5 µm. Representative images of (**D**) AM and (**E**) THP-1 stained with 2,7-dichlorofluorescein diacetate (DCFH-DA) for ROS levels (n = 5), Scale bars, 200 µm. (**F**) Quantitative statistical results of DCFH-DA-stained positive cells (n = 5). Pre-treatment with mitochondrial-specific ROS inhibitor Mito-TEMPO (50 nM, HY-112879, MCE) for 24 h followed by LPS and ATP, ELISA analysis of (**G**) IL-1β and (**H**) IL-18 in supernatants (n = 5). (**I**) Representative IF images for Nrf2 in THP-1 (n = 5), Scale bars, 20 µm and 5 µm (insets). (**J**) Quantitative statistical results of fluorescence intensity from Nrf2 with DAPI as reference (n = 5). (**K**) WB analysis of Nrf2 expression in THP-1 (n = 3). (**L**) Quantification of immunoblot grayscale values (n = 3). (**M**) Representative frozen lung tissue sections from LPS-induced mice (n = 5), stained with Dihydroethidium (DHE) for ROS detection, Scale bars, 100 µm. (**N**) Quantitative statistical results of DHE-stained positive cells (n = 5). In LPS-induced mouse lung tissue, (**O**) superoxide dismutase (SOD) activity, (**P**) catalase (CAT) activity, (**Q**) MDA concentrations were measured (n = 5). MDA: malondialdehyde; Nrf2: nuclear factor erythroid 2-related factor 2. The data represent one of at least three independent experiments. The values are shown as mean ± SD. *P < 0.05; **P < 0.01; ***P < 0.001; ****P < 0.0001.

Our previous studies revealed that GC-1 can restore epithelial mitochondrial function through the PINK1 pathway 18. The ratio of JC-10 aggregates to JC-10 monomers reflects the level of mitochondrial membrane potential. In THP-1 macrophages, GC-1 effectively reversed the LPS- and ATP-induced decrease in the mitochondrial membrane potential (Figure 3A-B), and normalized mitochondrial quantity and morphology (Figure 3C). These results suggest that GC-1 also contributes to the mitigation of mitochondrial damage in macrophages, which is a key determinant in elevated ROS levels. GC-1 alleviated the accumulation of ROS caused by LPS-induced mitochondrial damage, as evidenced by a decrease in DCFH-DA probe-positive cells and the MDA levels (Figure 3D-F, Figure S2A). Moreover, the mitochondrial-specific ROS inhibitor Mito-TEMPO reduced the production of IL-1β and IL-18 induced by LPS and ATP (Figure 3G-H), indicating that a decrease in ROS levels may inhibit macrophage pyroptosis. Further IF and WB investigations revealed that GC-1 significantly increased Nrf2 expression, thereby eliminating the inhibitory effect of LPS on this signal (Figure 3I-L). Consistently, in ALI model mice, GC-1 notably decreased ROS levels, as shown by reduced DHE staining (Figure 3M-N), increased SOD and CAT enzyme activities, and decreased MDA levels (Figure 3O-Q). Additionally, GC-1 increased Nrf2 expression levels in ALI model mice and its accumulation at the lesion site (Figure S2B-E). These results suggested that GC-1 activated the Nrf2 signaling and mitigated LPS-induced oxidative damage.

### GC-1 suppressed ROS-mediated pyroptosis in macrophages in an Nrf2-dependent manner

**Figure 4:**
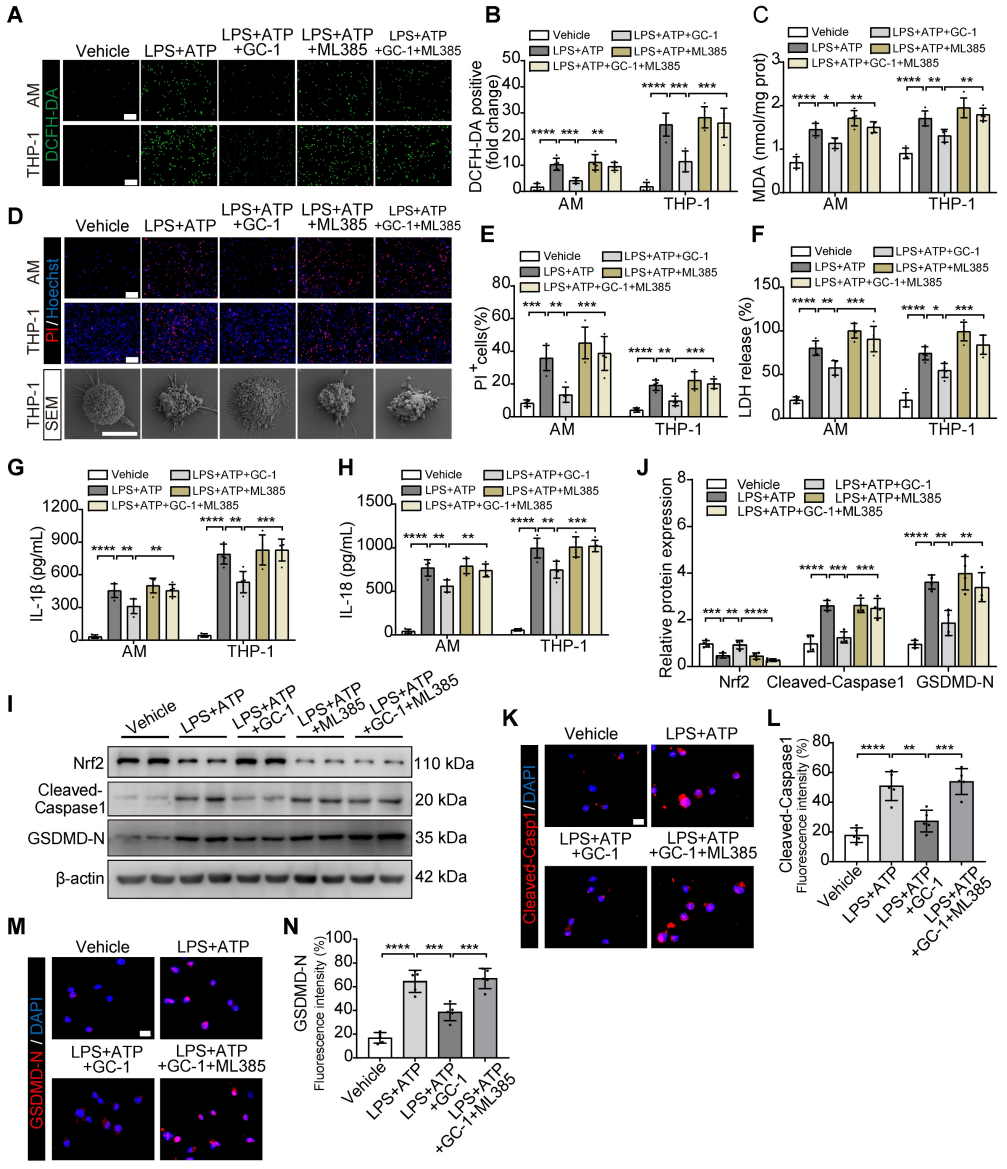
** The Nrf2-dependent pathway was utilized by GC-1 to inhibit ROS-induced pyroptosis in macrophages.** AM and THP-1 cells were pre-treated with ML385 (10 µM) for 12 h, followed by GC-1 (100 nM) for another 12 h, subsequent exposure to LPS (100 ng/mL) for 4 h, and finally induction with ATP (5 mM) for 45 min before sample collection. (**A**) Representative images of DCFH-DA staining (n = 5), Scale bars, 200 µm. (**B**) Quantitative statistical results of DCFH-DA-stained positive cells (n = 5). (**C**) Detection of malondialdehyde (MDA) concentrations (n = 5). (**D**) Representative images of PI and Hoechst33342 staining in AM and THP-1 cells (n = 5), Scale bars, 200 µm, as well as representative scanning electron microscopy (SEM) images of THP-1 (n = 3), Scale bars, 10 µm. (**E**) Quantitative statistical results of PI-stained positive cells (n = 5). (**F**) LDH cytotoxicity assay in supernatants, normalized to LPS+ ATP+ ML385-treated group (n = 5). ELISA analysis of (**G**) IL-1β and (**H**) IL-18 in THP-1 cell supernatants (n = 5). (**I**) Representative images of Western blot in THP-1 cells. (**J**) Immunoblot gray values statistical quantification (n = 4). Representative IF images of THP-1 for (**K**) Cleaved-Capase1 and (**M**) GSDMD-N (n = 5), Scale bars, 20 µm, and quantitative statistical results of fluorescence intensity from (**L**) Cleaved-Capase1 and (**N**) GSDMD-N with DAPI as reference (n = 5). GSDMD-N: n-terminal fragment of gasdermin D; LDH: lactate dehydrogenase; MDA: malondialdehyde; Nrf2: nuclear factor erythroid 2-related factor 2. The data represent one of at least three independent experiments. The values are shown as mean ± SD. *P < 0.05; **P < 0.01; ***P < 0.001; ****P < 0.0001.

Next, we pretreated AMs and THP-1 cells with the specific Nrf2 inhibitor ML385 and GC-1. ML385 effectively counteracted the protective effect of GC-1 on oxidative stress (Figure 4A-C). Additionally, the results confirmed that ML385 abolished the inhibitory effect of GC-1 on macrophage pyroptosis, as evidenced by an increase in PI-stained positive cells; swelling and ballooning of cells according to scanning electron microscopy (Figure 4D-E); and elevated levels of LDH, IL-1β, and IL-18 in the supernatants (Figure 4F-H). Further detection of the pyroptosis markers Cleaved-Caspase1 and GSDMD-N indicated that ML385 reversed the inhibitory effect of GC-1 on inflammasome activation, which restored the cleavage and activation of downstream Caspase1 and gasdermin D, as demonstrated by WB (Figure 4I-J) and IF analyses (Figure 4K-N). These results suggested that GC-1 suppressed inflammasome activation and pyroptosis in macrophages through an Nrf2-dependent mechanism.

### Inflammasome assembly was inhibited by GC-1 through the downregulation of ROS-activated p53 and ASC via Nrf2 signaling

**Figure 5:**
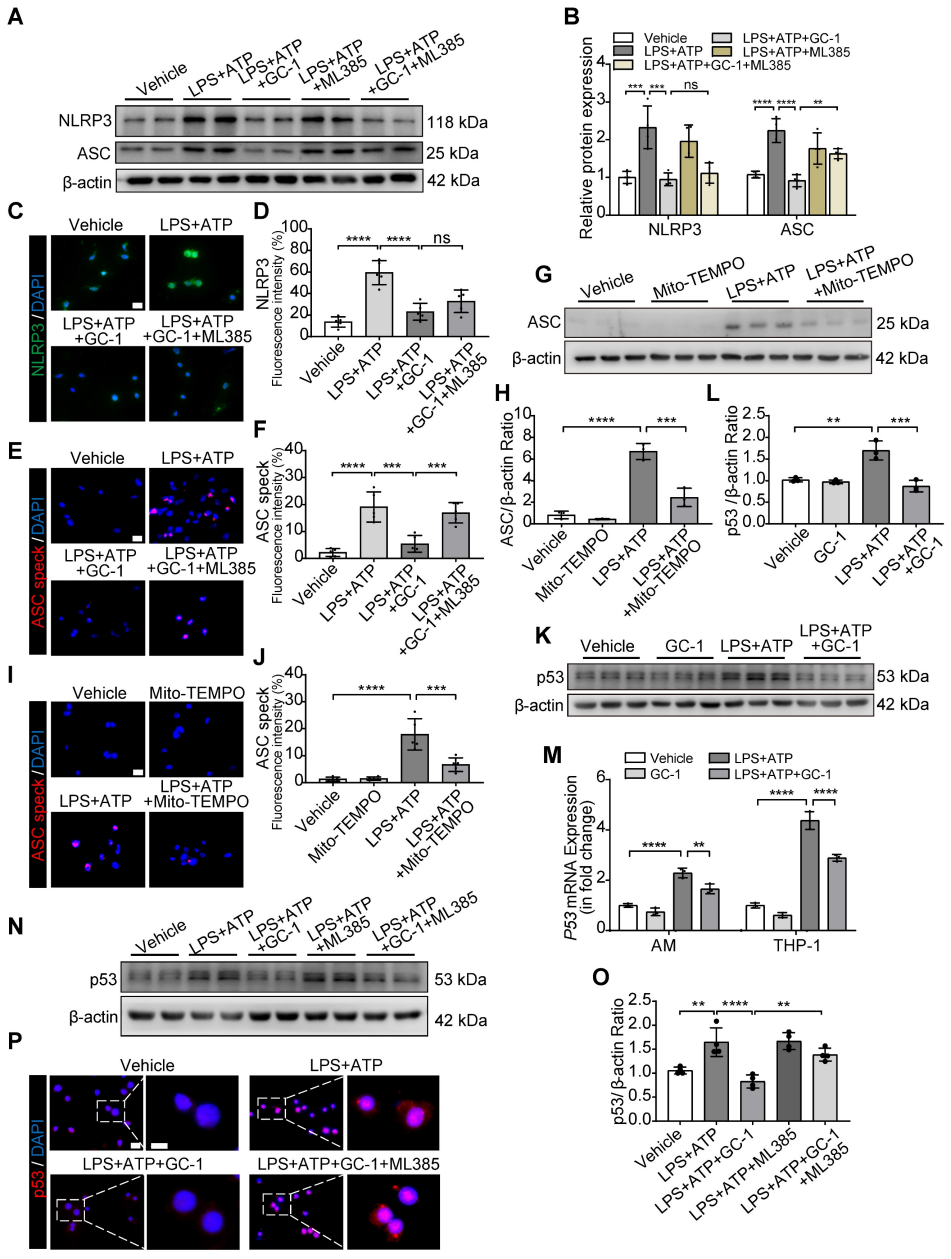
** GC-1 reduced p53 and ASC specks formation via Nrf2 signaling, inhibiting inflammasome assembly in macrophages.** (**A**) Representative images of Western blot for NLRP3 and ASC expression in THP-1. (**B**) Quantification of immunoblot grayscale values (n = 4). Representative IF images of (**C**) NLRP3 and (**E**) ASC speck (n = 5), Scale bars, 20 µm, and quantitative statistical results of fluorescence intensity from (**D**) NLRP3 and (**F**) ASC speck with DAPI as reference (n = 5). (**G**) Pre-treatment with Mito-TEMPO (50 nM) for 24 h followed by LPS and ATP, WB analysis of ASC expression in THP-1 (n = 3). (**H**) Quantification of immunoblot grayscale values (n = 3). (**I**) Representative IF images of ASC speck in Mito-TEMPO pre-treated THP-1 (n = 5), Scale bars, 20 µm. (**J**) Quantitative statistical results of fluorescence intensity from ASC speck with DAPI as reference (n = 5). (**K**) WB and (**M**) qRT-PCR analysis of p53 expression in THP-1 (n = 3). (**L**) Quantification of immunoblot grayscale values (n = 3). (**N**) Representative images of Western blot for p53 expression in THP-1 cells pretreated with ML385 (10 µM) for 12 h. (**O**) Quantification of immunoblot grayscale values (n = 4). (**P**) Representative IF images of p53 (n = 5), Scale bars, 20 µm and 10 µm (insets). ASC: apoptosis-associated speck-like protein containing a card; NLRP3: nlr family pyrin domain-containing 3. The data represent one of at least three independent experiments. The values are shown as mean ± SD. **P <0.01; ***P < 0.001; ****P < 0.0001; ns = not significant.

The assembly of the inflammasome is a critical process that triggers pyroptosis. The inhibitory effect of GC-1 on ASC was abolished by ML385, while the inhibitory effect on NLRP3 remained unaffected, as shown by WB (Figure 5A-B) and IF analyses (Figure 5C-F). Mito-TEMPO also markedly suppressed the ASC expression (Figure 5G-H) and ASC speck formation (Figure 5I-J) induced by LPS and ATP; this indicated that GC-1 reduced ROS-mediated ASC recruitment and oligomerization to prevent inflammasome assembly through a Nrf2-dependent mechanism. Furthermore, the specific PPARγ inhibitor GW9662 eliminated the inhibitory effect of GC-1 on NLRP3 (Figure S3A-B), which suggested that GC-1 may inhibit NLRP3 expression associated with the PPARγ-NF-κB pathway. Further investigation revealed that GC-1 significantly reduced the expression of p53 (a transcriptional regulator of ASC) induced by LPS and ATP (Figure 5K-M), whereas ML385 abolished the inhibitory effect of GC-1 on p53 expression (Figure 5N-O) and nuclear translocation (Figure 5P). These findings revealed that GC-1 inhibited p53 expression through Nrf2 signaling and decreased ASC recruitment and oligomerization. Overall, these results indicated that GC-1 reduced ROS-activated p53 and ASC expression by activating Nrf2, thereby preventing NLRP3 inflammasome assembly in macrophages.

### Inhibition of Nrf2 aggravated pyroptosis and lung injury in ALI model mice

**Figure 6:**
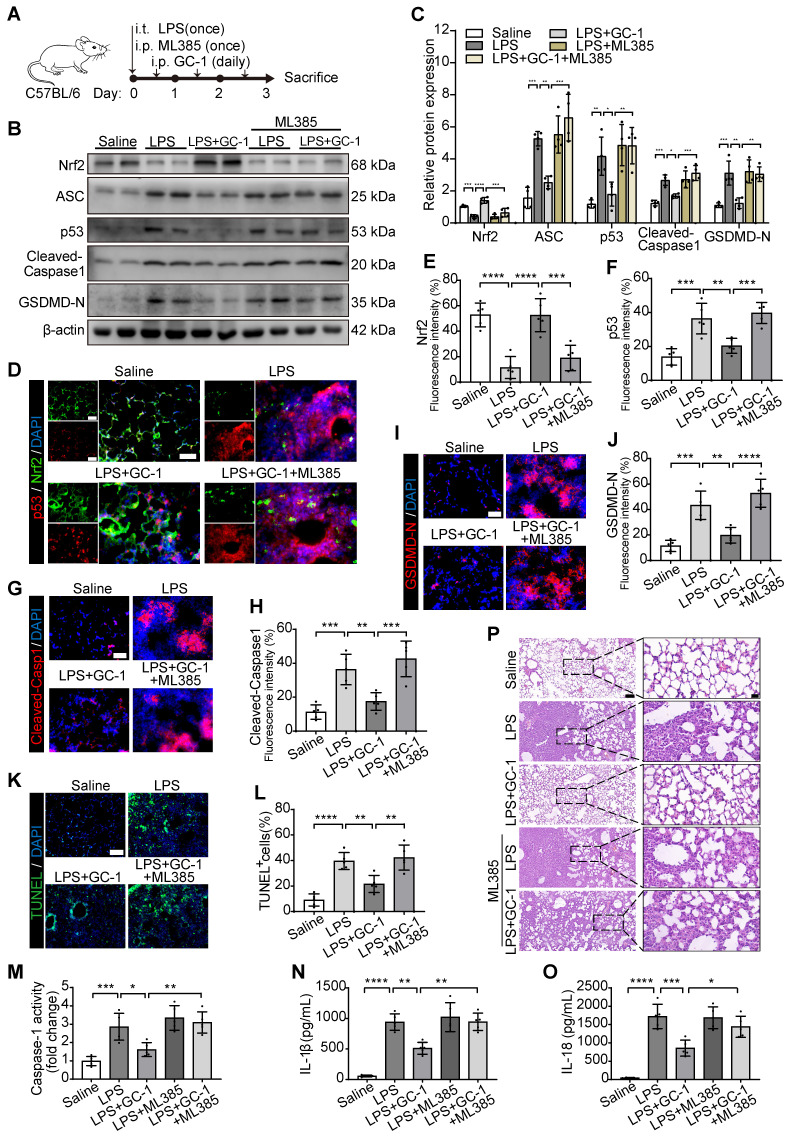
** Inhibition of Nrf2 promoted oxidative stress, pyroptosis and lung injury in ALI mice.** C57BL/6 mice were treated with LPS and ML385 (30 mg/kg body weight), as described in methods. (**A**) Schematic representation of the experiment. (**B**) Representative images of Western blot in mouse lung tissues. (**C**) Quantification of immunoblotting grayscale values (n = 4). Representative frozen lung tissue sections of mice stained for (**D**) p53 and Nrf2, (**G**) Cleaved-Capase1, and (**I**) GSDMD-N IF images (n = 5), Scale bars, 50 µm, and quantitative statistical results of fluorescence intensity from (**E**) Nrf2, (**F**) p53, (**H**) Cleaved-Capase1 and (**J**) GSDMD-N with DAPI as reference (n = 5). (**K**) Representative sections of mice stained with TUNEL (n = 5), Scale bars, 100 µm. (**L**) Quantitative statistical results of TUNEL-stained positive cells (n = 5). (**M**) Measurement of Caspase-1 activity, normalized to saline group (n = 5). ELISA analysis of (**N**) IL-1β and (**O**) IL-18 levels in mouse BAL fluid (n = 5). (**P**) Representative paraffin-embedded lung sections (n = 5), H&E staining, Scale bars, 100 µm and 20 µm (insets). ASC: apoptosis-associated speck-like protein containing a card; GSDMD-N: n-terminal fragment of gasdermin D; Nrf2: nuclear factor erythroid 2-related factor 2. The data represent one of at least three independent experiments. The values are shown as mean ± SD. *P < 0.05; **P < 0.01; ***P < 0.001; ****P < 0.0001.

We further validated the *in vitro* results via the intraperitoneal injection of ML385 into LPS-induced ALI model mice (Figure 6A). ML385 significantly inhibited the scavenging effect of GC-1 on ROS, which led to an increase in DHE-positive staining and MDA levels in lung tissues (Figure S4A-B). Additionally, the administration of ML385 abrogated the stimulatory effect of GC-1 on Nrf2 and the inhibitory effects of GC-1 on p53 and ASC in mice with LPS-induced ALI, thereby negating the suppressive effect of GC-1 on pyroptosis (Figure 6B-C). GC-1-induced Nrf2 accumulation in the lung lesion area was decreased by treatment with ML385 (Figure 6D-E), whereas there was an increase in the accumulation of p53, Cleaved-Caspase1, and GSDMD-N (Figure 6F-J). ML385 also increased TUNEL-positive staining (Figure 6K-L), pulmonary Caspase-1 enzyme activity (Figure 6M), and BAL IL-1β and IL-18 concentrations (Figure 6N-O). These findings suggested that inhibiting Nrf2 expression exacerbated the severity of pyroptosis in ALI model mice. Consistently, ML385 also abolished the protective effect of GC-1 on lung tissue damage in ALI model mice, resulting in aggravated pathological injury (Figure 6P, Figure S4C-D). In summary, GC-1 exerted a therapeutic effect on ALI model mice by inhibiting oxidative damage and pyroptosis through Nrf2 signaling.

## Discussion

In this study, we assessed the therapeutic effect of the thyromimetic GC-1 on LPS- or HCl-induced ALI in mice. GC-1 significantly improved inflammation and pulmonary damage in ALI model mice, as indicated by tissue pathology, ROS production and pyroptosis rates. Further research revealed that GC-1 may mitigate the oxidative stress induced by mitochondrial damage through the Nrf2 signaling pathway, thereby reducing the expression of ROS-activated p53 and ASC. This inhibited NLRP3 inflammasome assembly and pyroptosis, which ultimately decreased cell membrane rupture and the production of inflammatory factors. These results suggested that GC-1 may serve as an anti-inflammatory and antioxidant drug that protects against ALI by inhibiting macrophage oxidative stress and pyroptosis.

Many studies have shown that THs are crucial for maintaining cellular homeostasis under stress conditions, including nonalcoholic fatty liver disease, systolic heart failure, chronic kidney disease, and the SARS and COVID-19 epidemics 25. Due to the shared origin of the lung and thyroid from Nkx2.1 cells of the endoderm during embryonic development 26, the regulation of pulmonary diseases by THs is highly regarded, despite the controversial and cautious history of TH therapy 22, 27. This study demonstrated that GC-1 inhibited macrophage pyroptosis through the Nrf2-p53-ASC pathway, effectively alleviating lung injury in mice. This research further expands on our previous findings on the ability of T_3_ and GC-1 to mitigate pulmonary fibrosis and lung injury by improving epithelial mitochondrial function 18, 19. Furthermore, GC-1 avoids the side effects of cardiac thyrotoxicosis and has shown promising therapeutic effects and safety in phase I clinical trials 21, 28. Therefore, further research on the therapeutic effects of GC-1 and the mechanisms of interaction between the endocrine and immune systems is promising.

The global effects of COVID-19 have highlighted the importance of the use of precision medicine for treating ARDS 4, 29. The complexity of the pathogenesis of ARDS involves multiorgan damage and dysregulation of inflammatory pathways 1. Inflammation plays a significant role in ALI/ARDS, and targeting inflammation remains a challenge in clinical practice 30. Increasing evidence suggests that soluble cell death, such as pyroptosis and necroptosis, is a powerful trigger of inflammation and disease 6. Severely ill COVID-19 patients exhibited extreme pyroptosis in monocytes with high levels of Cleaved-Caspase1 and IL-18 in their serum 31. Our study revealed that LPS and ATP induced mitochondrial damage in macrophages, leading to ROS accumulation, pyroptosis and the release of IL-1β and IL-18. GC-1 and the ROS inhibitor Mito-TEMPO effectively inhibited pyroptosis and lung injury in mice. Consistent with our results, Hsu *et al.* reported that the lipid peroxidation product prevented pyroptosis and inflammation in mouse macrophages and human peripheral blood monocytes 32. Jiao *et al.* also demonstrated that inhibiting neutrophil extracellular vesicles reduced macrophage pyroptosis and lung tissue damage in mice 33. These results suggest that inhibiting pyroptosis to reduce the release of inflammatory cytokines is effective for preventing ALI/ARDS.

The inflammasome is an innate immune defense mechanism that protects against pathogen infections and has emerged as a key regulatory factor in inflammation-related diseases 7. ASC, an adaptor protein in the inflammasome complex, connects different sensors to activate caspase1 and trigger pyroptosis as well as the release of inflammatory cytokines 8, 9.

After pyroptosis, ASC oligomers aggregate outside the cell, continuously promoting IL-1β maturation and further amplifying the inflammatory response 34. Thus, the humanized anti-ASC antibody IC-100, the camel-derived VHH_ASC_ nanobody, and the small-molecule inhibitor MM01 have been developed for ASC-dependent inflammasome-mediated diseases 35-38. Here, we also confirmed that GC-1 effectively reduced ASC recruitment and oligomerization in macrophages. Our findings are supported by results from murine models of gouty arthritis, ulcerative colitis, and cytomegalovirus infection, which suggest that targeting ASC can inhibit the activation of the NLRP3 and AIM2 inflammasome and alleviate inflammation 39. Additionally, studies have shown that GC-1 promotes oligodendrocyte progenitor cell differentiation and stimulation of myelin repair in the brain and spinal cord 40. Our results indicate that the anti-inflammatory properties of GC-1 present a new perspective for the treatment of neurodegenerative diseases associated with neuroinflammation, such as multiple sclerosis 35.

Mitochondrial damage and ROS accumulation exacerbate inflammatory damage in ALI/ARDS 11. Nrf2 plays a crucial protective role in regulating antioxidant, anti-inflammatory, and detoxification responses. We found that GC-1 inhibited ROS-induced NLRP3 inflammasome assembly in an Nrf2-dependent manner. Mito-TEMPO had effects similar to those of GC-1, whereas ML385 abolished the antioxidant effects of GC-1. Our findings are further supported by studies from ischemia/reperfusion and sepsis-induced ALI mouse models, which demonstrated that targeting Nrf2 reduces oxidative stress and inflammatory damage in mice 16. Currently, NRF2 agonists such as sulforaphane and bardoxolone methyl have entered clinical trials and have shown promising cellular protection and anti-inflammatory effects 41. These results highlight the potential of GC-1 as an antioxidant in ALI/ARDS.

The transcription factors p53 and Nrf2 regulate oxidative stress in different ways. p53 plays a dual role in promoting both cell survival and apoptosis under conditions of oxidative stress. Excessive accumulation of ROS stimulates p53 expression, leading to apoptosis to eliminate the damaged cells 14. Research on mouse models of acute liver failure and bone marrow suppression has confirmed that Nrf2 activation enhances the antioxidant capacity of mice, thereby inhibiting p53-mediated cell apoptosis 42. Obese mice models have also shown that the inhibition of fatty acid binding protein 4 regulates the activation of the NLRP3 inflammasome and pyroptosis through the p53-ASC pathway 43. We consistently found that LPS induced high levels of ROS, which upregulated the expression of p53 and its downstream target gene ASC to trigger pyroptosis. GC-1 activated Nrf2 to remove excessive ROS, reduced p53 expression, and inhibited inflammasome activation. Therefore, GC-1 may reduce oxidative stress and pyroptosis in macrophages via the Nrf2-p53-ASC signaling pathway.

In summary, this study demonstrated that GC-1, a promising drug candidate for ALI/ARDS and other inflammation-related diseases, effectively alleviated inflammation and pulmonary damage in ALI model mice. Further research suggested that GC-1 may reduce the expression of ROS-activated p53 and ASC recruitment and oligomerization, inhibiting NLRP3 inflammasome assembly and pyroptosis via the Nrf2-p53-ASC signaling. This study increases our understanding of the novel role of GC-1 in pulmonary inflammation, revealing the mechanisms involved in endocrine and immune system interactions. This exploration is important for the development of personalized medicine for treating endocrine and pulmonary diseases in clinical practice.

## Materials and methods

### Animal experiment and experimental design

8-week-old C57BL/6 mice of either gender were purchased from Beijing Charles River Laboratory Animal Technology Co., Ltd. (Beijing, China) and housed in specific pathogen-free conditions. The mice were randomly divided into groups (10 mice per group) and treated as previously described 18. For LPS or HCl induced ALI, on day 0, mice were anesthetized by inhaling 40% isoflurane (RWD, Shenzhen, China) diluted in 1,2-Propanediol (Aladdin, Shanghai, China). A single dose of 5 mg/kg LPS O111:B4 (Sigma-Aldrich, Missouri, USA) dissolved in 50 μL saline, or 50 μL HCl (0.1 M, pH 1.0) was administered intratracheally. 12 h later, mice were intraperitoneally injected with 100 μg/kg GC-1 (Sigma-Aldrich) in 0.2 mL saline daily for three days. On the 3rd day, mice were euthanatized with an intraperitoneal injection of 20% Ethyl Carbamate (800 mg/kg, Sigma-Aldrich). lung tissues and BAL fluid were collected and frozen for further tests. In the study of Nrf2 inhibitor, a single intratracheal administration of LPS was performed. Simultaneously, a one-time intraperitoneal injection of 30 mg/kg ML385 (MCE, Shanghai, China) in 0.2 mL saline was administered. After 12 h, mice were subjected to daily intraperitoneal injections of GC-1 for three consecutive days. Control group mice received the same volume of saline.

The animal care and handling followed the guidelines of the Henan Normal University Institutional Animal Care and Use Committee (IACUC, SMKX-2118BS1018), in accordance with the standards set by the Animal Behavior Society and national regulations.

### Cell culture

THP-1 cell line was purchased from Procell (CL-0233, Pricella Life Science&Technology Co.,Ltd, Wuhan, China) and cultured in RPMI-1640 medium (Procell, PM150110) with 10% fetal bovine serum (v/v), 100 U/mL penicillin, and 100 mg/L streptomycin (HY-K1006, MCE) at 37 °C with 5% CO_2_. Mouse AMs were isolated from BAL fluid, resuspended in complete RPMI-1640 medium, incubated at 37 °C with 5% CO_2_ for 4-6 h, then washed with PBS to remove non-adherent cells. Adherent cells were identified using immunofluorescence and Diff-Quik staining methods. Myco-Lumi™ Luminescent Mycoplasma Detection Kit (C0298S, Beyotime, Shanghai, China) was used to test for mycoplasma infection in cells.

### Mouse bronchoalveolar lavage (BAL) fluid

Following euthanatized with 20% Ethyl Carbamate, a tracheostomy was performed to expose the tracheae. A 20 G (1.1 mm) indwelling needle was inserted into the upper part of the trachea, and 0.8 mL of pre-cooled saline was slowly injected using a 1mL syringe. This process was repeated three times, and the recovered lavage fluid volume was recorded.

### Hematoxylin and eosin (H&E) staining and lung injury scores

After deparaffinization and rehydration, 4 μm paraffin-embedded mouse lung tissue sections were stained with hematoxylin for nuclear labeling and eosin for cytoplasmic staining (Beyotime). Subsequent dehydration and mounting were carried out before capturing images using an optical microscope. According to the American Thoracic Society (ATS) lung injury scoring system, 20 random fields (400× magnification) were independently scored in a blinded manne for each slide.

### BAL fluid protein concentration assay

The Bicinchoninic Acid (BCA) Protein Concentration Assay Kit (PC0020, Solarbio, Beijing, China) was used to determine the protein concentration in mouse BAL fluid (BALF). Following the kit instructions, BCA working solution was prepared and protein standards were diluted. Mouse BALF was collected as described before and centrifuged at 2000 rpm for 5 min. Then, 20 μL supernatant and 200 μL BCA working solution were added to a 96-well plate and incubated at 37 °C for 30 min. The absorbance value at 562 nm was measured using a Bio-Rad enzyme reader. Finally, BALF protein concentration was calculated, based on the protein standard curve.

### BALF white blood cells (WBC) concentration assay

As previously mentioned 44, the automated hematology analyzer (DxH 500, Beckman Coulter, California, USA) was used for the detection of WBC concentration from mouse BALF. In brief, 0.2 mL mouse BALF was mixed with an appropriate amount of saline to ensure a suitable detection range. Subsequently, the tests were performed according to the instructions provided by the manufacturer.

### Enzyme-linked immunosorbent assay (ELISA)

According to the instructions of the kits (Solarbio, SEKM-0002/SEKH-0002/SEKH-0028/SEKM-0019), ELISA was performed to detect the concentrations of IL-1β and IL-18 in mouse BALF, AM and THP-1 cell supernatants. Briefly, 100 μL mouse BALF and cell supernatant were appropriately diluted and added to microplate wells. The plate was then incubated at 37 °C for 90 min. After washing, a series of solutions including biotinylated antibody working solution, enzyme conjugate working solution, and chromogenic substrate were sequentially added according to the instructions for further incubation. Finally, 50 μL termination solution was added and the OD value was immediately measured at 450 nm using a Bio-Rad enzyme reader. The concentrations of IL-1β and IL-18 were calculated based on the standard curve.

### TUNEL assay

The one-step TUNEL *in situ* apoptosis kit (Elabscience® Biotechnology, E-CK-A320) was utilized for the assessment of cell death in frozen sections of mouse lung tissue. In short, the frozen sections were fixed with 4% paraformaldehyde for 60 min and subsequently incubated with 0.5% Triton X-100 (MCE, HY-Y1883A) at room temperature for 5 min. Following this, they were treated with 50 μL TUNEL assay solution and incubated in the dark at 37 °C for 60 min. After sealing the slices using an anti-fluorescence quenching solution, images were captured utilizing a Zeiss fluorescence microscope.

### SOD and CAT activities assay

According to the instructions of the SOD and CAT activity assay kit (Solarbio, BC4785/BC0175), the colorimetric method was employed to detect the changes in SOD and CAT activities in mouse lung tissue samples, evaluating the antioxidant capacity. In brief, 0.1 g mouse lung tissue was homogenized with 1 mL extract in an ice bath. The mixture was then centrifuged at 10000 rpm for 10 min at 4 °C. Subsequently, 20 μL supernatant was incubated at 37/25 °C for 30/10 min following the instructions. Finally, a Bio-Rad enzyme analyzer was used to measure absorbance at 560/405 nm for calculating SOD/CAT activities based on the instructions' formulas.

### ROS levels analysis

The ROS levels in frozen sections of mouse lung tissue were assessed using the ROS detection kit (Beyotime, S0063). Following incubation with a 5 uM DHE fluorescent probe at 37 °C in the absence of light for 30 min, the nuclei were counterstained with DAPI (MCE, HY-D1738) and imaged under a Zeiss fluorescence microscope. Additionally, the ROS levels in mouse AMs and THP-1 cells were determined utilizing the ROS detection kit (Solarbio, CA1410), where cells were treated with a 10uM DCFH-DA fluorescent probe at 37 °C in the dark for 20 min. Subsequently, after washing, images were captured using a Zeiss fluorescence inverted microscope.

### Mitochondrial membrane potential

According to the instructions of JC-10 mitochondrial membrane potential fluorescent probe (Solarbio, J8050), 0.5 ml of JC-10 working solution was mixed with an appropriate amount of THP-1 cells and incubated at 37 °C for 20 min. The supernatant was discarded by centrifugation at 2000 rpm, and after washing with JC-10 staining buffer. Flow cytometry was performed to detect mitochondrial membrane potential, assessing the degree of mitochondrial damage.

### Quantitative real-time PCR (qRT-PCR)

Total RNA was extracted using the miRNeasy Mini kit (QIAGEN, Dusseldorf, Germany) and reverse transcribed using Prime Script reverse transcriptase (Promega, Wisconsin, USA). qRT-PCR was carried out using qPCR SYBR Green Master Mix (11201ES08, Yeasen, Shanghai, China) according to the instructions. β-actin was used as a reference, with each sample run in triplicate. The primer sequences used in this study were: ACTB-F, CATGTACGTTGCTATCCAGGC, ACTB-R, CTCCTTAATGTCACGCACGAT; p53-F, CAGCACATGACGGAGGTTGT, p53-R, TCATCCAAATACTCCACACGC.

### Western Blot (WB)

Protein extraction from lung tissues and cells was performed as previously described. 20 mg protein samples were separated by 8-12% SDS-PAGE gel and transferred to a PVDF membrane (Millipore, Massachusetts, USA). After blocking, the membrane was incubated overnight at 4 °C with specific primary antibodies (anti-β-actin (Affinity, T0022), anti-Nrf2 (Affinity, AF0639), anti-p53 (Proteintech, 60283-2-Ig), anti-NLRP3 (Affinity, DF7438), anti-ASC (Affinity, DF6304), anti-Caspase1 (Affinity, AF5418), anti-Cleaved-Caspase1 (Affinity, AF4005), anti-GSDMD-N (Affinity, AF4012)), followed by incubation with secondary antibodies (Abcam, ab205718, ab6789) at room temperature for 2 h. Protein bands were visualized using a chemiluminescence system (Bio-Rad, USA), with β-actin as a reference.

### Immunofluorescence

6 μm frozen sections of mouse lung tissue were fixed with 4% paraformaldehyde for 10 min, treated with 0.3% Triton X-100 for 5 min, and then incubated overnight at 4 °C with specific primary antibodies (anti-NLRP3 (Bioss, bs-41293R), anti-Cleaved-Caspase1 (Affinity, AF4005), anti-GSDMD-N (Affinity, AF4012), anti-Nrf2 (Affinity, AF0639), anti-p53 (Proteintech, 60283-2-Ig)). Following incubation with fluorescent secondary antibodies (Abcam, ab150113, ab150079) at room temperature for 1 h in the dark, cell nuclei were stained with DAPI and images were captured using a Zeiss fluorescence microscope.

### Scanning electron microscopy (SEM)

After centrifugation of THP-1 cells treated with the above method, the samples were fixed using a fixative and sent to Wuhan servicebio technology Co., Ltd. (Hubei, China) for SEM analysis. Briefly, the cells were washed three times with 0.1 M PB (pH 7.4). Subsequently, they were transferred into a solution containing 1% OsO_4_ in 0.1 M PB (pH 7.4) and kept at room temperature for 1-2 h. After undergoing gradient dehydration, the samples were placed onto cover slips and dried using a Critical Point Dryer. Specimens were attached to metallic stubs using carbon stickers and sputter-coated with gold for 30 s before being imaged using a scanning electron microscope (HITACHI, SU8100).

### Propidium Iodide (PI) staining

The integrity of cell membrane was evaluated by staining mouse AMs and THP-1 cells with PI and Hoechst33342 using cell apoptosis and necrosis detection kit (Beyotime, C1056). A mixture of 5 μL Hoechst and 5 μL PI staining solution with the appropriate number of cells was incubated at 4 °C for 30 min, followed by imaging under a Zeiss fluorescence microscope.

### Lactate dehydrogenase (LDH) cytotoxicity assay

The LDH activity in the supernatant of AMs and THP-1 cells was assessed using the LDH cytotoxicity detection kit (Beyotime, C0016) to evaluate the extent of cell membrane disruption. Briefly, 120 μL cell supernatant and 60 μL LDH assay working solution were combined in a 96-well plate and incubated at room temperature for 30 min in darkness. Subsequently, absorbance at 490 nm was measured using a Bio-Rad microplate reader, and the results were normalized against the LPS-treated group.

### Caspase-1 activity assay

Caspase-1 activity assay kit (Solarbio, BC3810) was employed for mouse lung tissues, AMs, and THP-1 cells to evaluate the activation of inflammasome. In brief, lung tissues or cells were thoroughly homogenized and centrifuged at 15000 rpm for 15 min at 4 °C. 50 μL supernatant was collected and incubated at 37 °C for 120 min following the provided instructions. The absorbance at 405 nm was measured using a Bio-Rad plate reader, and results were normalized to the control group.

### Malondialdehyde (MDA) content assay

The MDA content in mouse lung tissue, AMs and THP-1 cells was determined using the MDA content detection kit (Solarbio, BC0025) to assess the extent of oxidative damage. An appropriate amount of extract was added to the lung tissue and cells, followed by thorough homogenization in an ice bath and centrifugation at 10000 rpm for 10 min at 4 °C. 100 μL supernatant was then subjected to a water bath at 100 °C for 60 min according to the instruction. After cooling, the samples were further centrifuged at 2000 rpm for 10 min, and the absorbance values at 450/532/600 nm were measured using a Bio-Rad enzyme reader. Finally, the MDA content was calculated using the formula provided by the manufacturer.

### Statistical analyses

The data represented one of at least three independent experiments and were analyzed using GraphPad Prism 7.0 (GraphPad Software, Inc.). The software serial number was GPS-0320559-LFUL-95242. One-way analysis of variance with Tukey's multiple comparison test was employed for comparisons among multiple groups. All data were presented as mean ± standard deviation (SD), with statistical significance considered at *P* < 0.05. The sample size was not predetermined using statistical methods. Mice were randomly assigned to experimental groups, and there were no exclusion criteria for animals.

## Supplementary Material

Supplementary figures.

## Abbreviations

ALI: acute lung injury
AMs: alveolar macrophages
ARDS: acute respiratory distress syndrome
ASC: apoptosis-associated speck-like protein containing a card
BAL: bronchoalveolar lavage
DHE: Dihydroethidium
GSDMD-N: n-terminal fragment of gasdermin D
LDH: lactate dehydrogenase
MDA: malondialdehyde
NLRP3: nlr family pyrin domain-containing 3
Nrf2: nuclear factor erythroid 2-related factor 2
ROS: reactive oxygen species
THs: thyroid hormones
TRβ1: thyroid hormone receptor β1
