# Supplementary material for: Inhibition of macrophage inflammasome assembly and pyroptosis with GC-1 ameliorates acute lung injury
Source: Theranostics. 2025 Jan 20;15(6):2360–74. doi: 10.7150/thno.101866 (PMC11840730; doi:10.7150/thno.101866)
Supplement: Supplementary file 1 — Supplementary figures. [file thnov15p2360s1.pdf]

Supplementary Materials

Title

Inhibition of macrophage inflammasome assembly and pyroptosis with GC-1 ameliorates acute lung injury

Authors

Bin Li, Jingyi Liu, Wanyu He, Yanlin Zhou, Man Zhao, Cong Xia, Xiaoyue Pan, Zhihua Ji, Ruoyu Duan, Hui Lian, Kai Xu, Guoying Yu*, Lan Wang*

This file includes: Figure S1-4

Supplementary figures

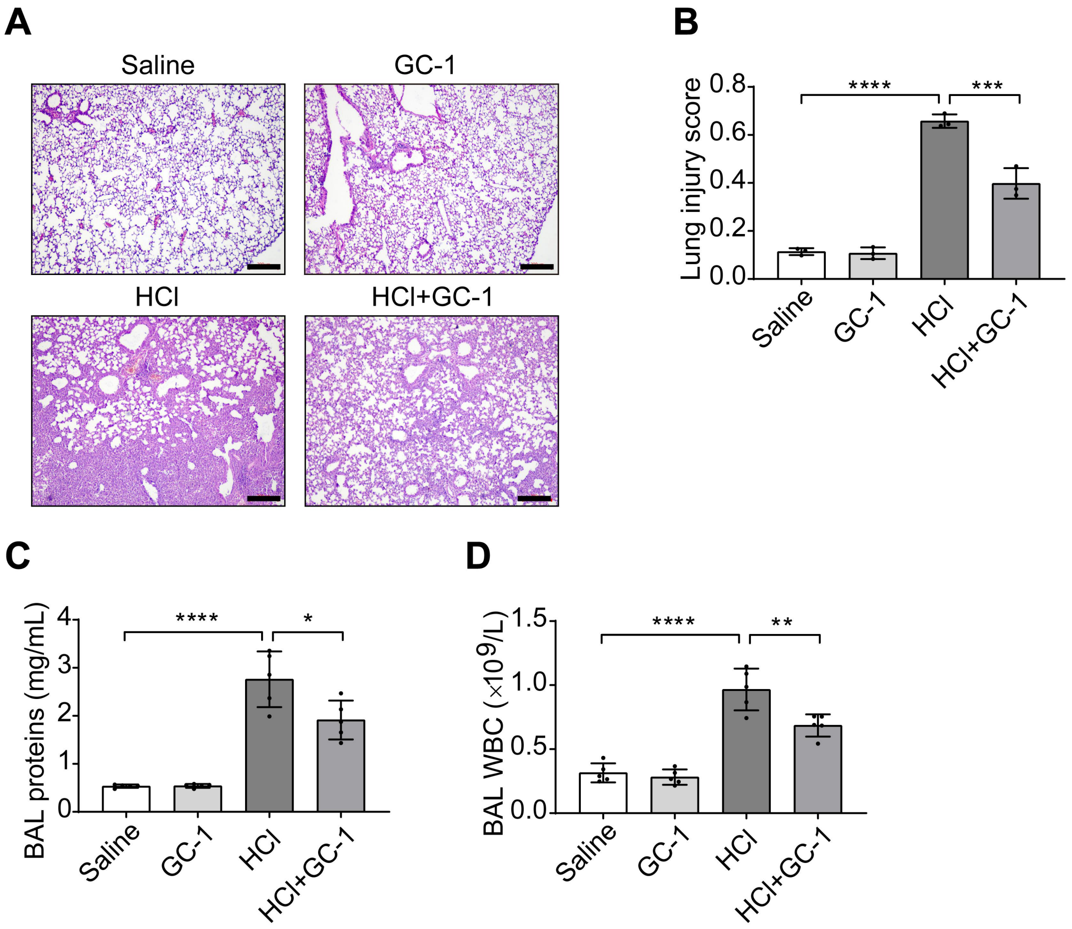

Figure S1. GC-1 alleviated lung injury induced by HCl in mice. The treatment of C57BL/6 mice was the same as in Figure 1. (**A**) Representative paraffin sections from HCl-induced mice (n = 3), H&E staining, Scale bars, 200 μm. (**B**) Lung injury score of HCl-induced ALI according to the ATS lung injury scoring system (n = 3). The levels of (**C**) BAL protein content and (**D**) BAL WBC concentration were measured in HCl-induced mice (n = 5). BAL: bronchoalveolar lavage. The data represent one of at least three independent experiments. The values are shown as mean ± SD. **P* < 0.05; ***P* < 0.01; ****P* < 0.001; *****P* < 0.0001.

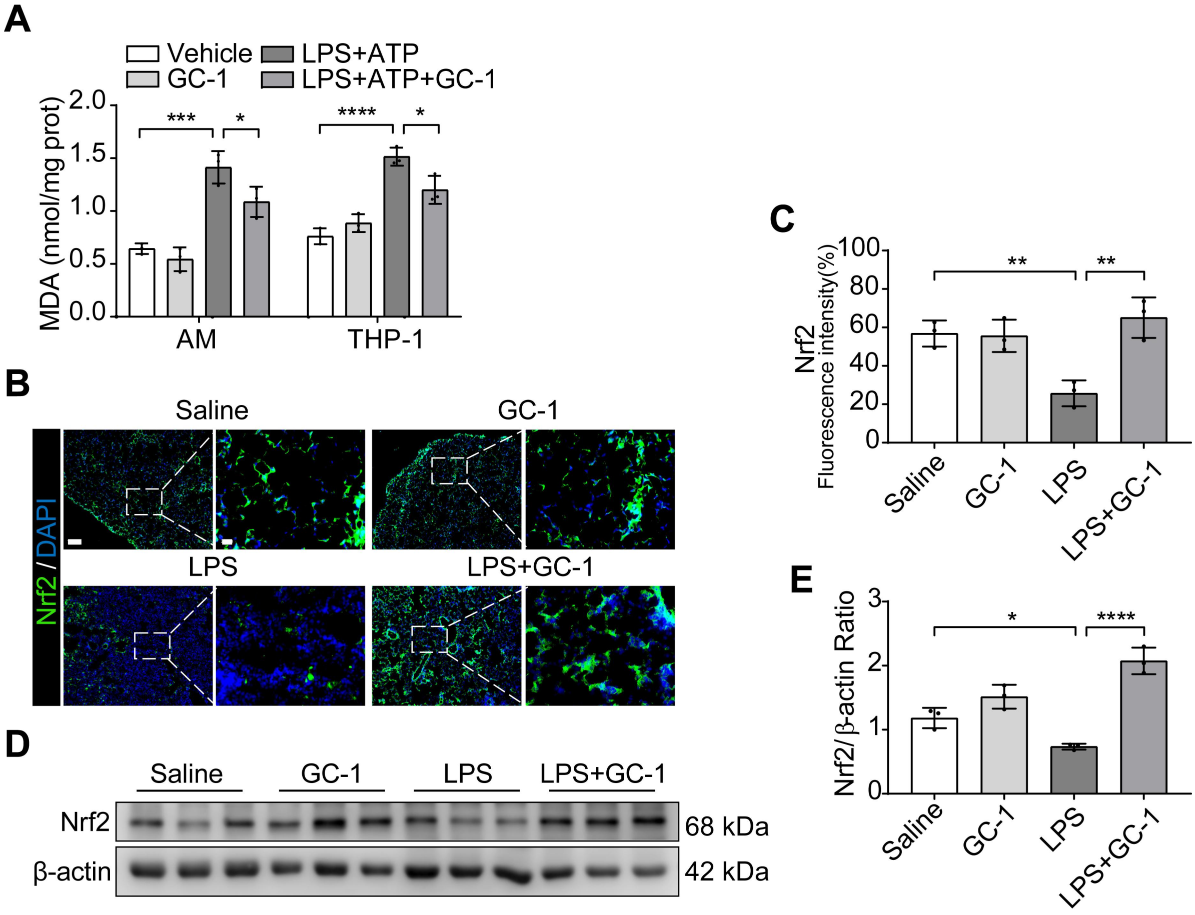

Figure S2. GC-1 mitigated oxidative damage in vivo and in vitro. (**A**) Detection of MDA concentrations in AM and THP-1 (n = 3). (**B**) Representative IF images in mice (n = 3), Scale bars, 100 μm and 20 μm (insets). (**C**) Quantitative statistical results of fluorescence intensity from Nrf2 with DAPI as reference (n = 3). (**D**) Western blot analysis of Nrf2 expression in lung tissue (n = 3). (**E**) Quantification of immunoblot grayscale values (n = 3). The data represent one of at least three independent experiments. MDA: malondialdehyde; Nrf2: nuclear factor erythroid 2-related factor 2. The values are shown as mean ± SD. **P* < 0.05; ***P* < 0.01; ****P* < 0.001; *****P* < 0.0001; ns = not significant.

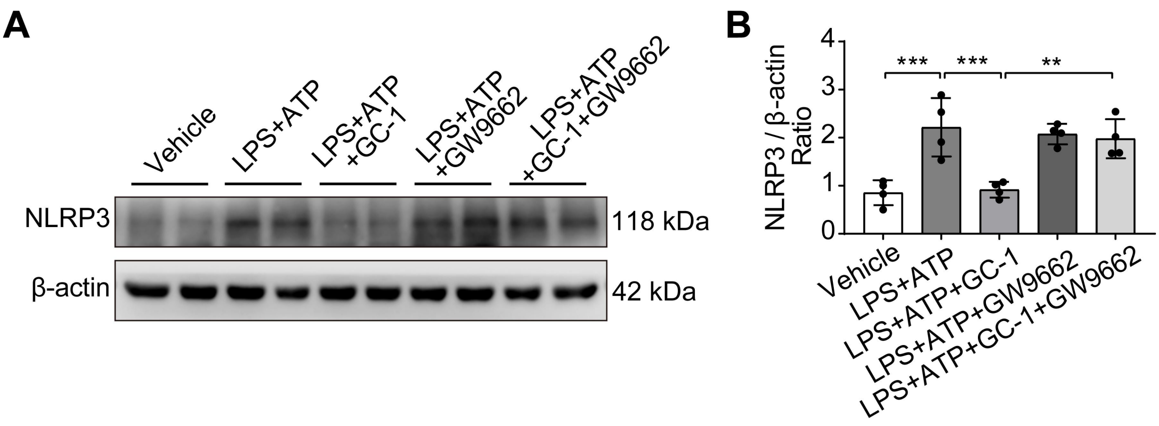

Figure S3. Inhibition of PPARγ reversed the effect of GC-1 on NLRP3. THP-1 cells were pretreated with GC-1 (100 nM) and/or the PPARγ inhibitor GW9662 (5 μM) for 12 h, followed by treatment with LPS and ATP. (**A**) Representative images of Western blot for NLRP3 in THP-1 cells. (**B**) Quantitative analysis of immunoblot grayscale values (n = 4). NLRP3: nlr family pyrin domain-containing 3. The data represent one of at least three independent experiments. The values are shown as mean ± SD. *$P < 0.05$; **$P < 0.01$; ***$P < 0.001$; ****$P < 0.0001$.

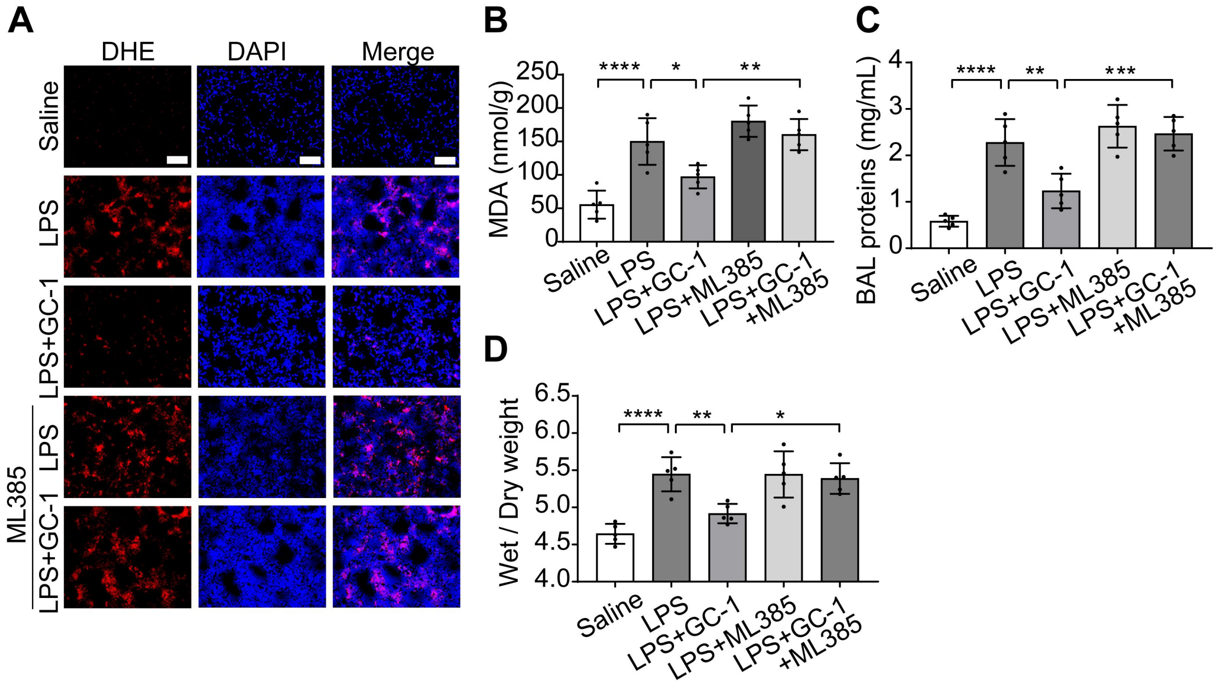

Figure S4. Inhibition of Nrf2 exacerbated oxidative damage and lung injury in ALI mice. (**A**) Representative frozen sections of lung tissue (n = 3), stained with DHE, Scale bars, 100 μm. (**B**) Measurement of MDA concentrations in lung tissue (n = 5). (**C**) BAL protein content and (**D**) lung wet/dry weight ratio were measured in LPS-induced mice (n = 5). The data represent one of at least three independent experiments. BAL: bronchoalveolar lavage; DHE: Dihydroethidium; MDA: malondialdehyde. The values are shown as mean ± SD. *$P < 0.05$; **$P < 0.01$; ***$P < 0.001$; ****$P < 0.0001$.
